# Geographic distribution of general industry payments to advanced-practice clinicians

**DOI:** 10.1093/haschl/qxad011

**Published:** 2023-06-20

**Authors:** Qiwei Wilton Sun, Joseph J Cavallo, Howard P Forman

**Affiliations:** Yale School of Medicine, New Haven, CT 06510, United States; Department of Radiology and Biomedical Imaging, Yale School of Medicine, 789 Howard Avenue, Tompkins East 204, New Haven, CT 06510, United States; Department of Radiology and Biomedical Imaging, Yale School of Medicine, 789 Howard Avenue, Tompkins East 204, New Haven, CT 06510, United States; Yale School of Management, New Haven, CT 06510, United States; Health Policy and Management Department, Yale School of Public Health, New Haven, CT 06510, United States

**Keywords:** open payments, advanced-practice clinicians, scope of practice

## Abstract

Following the recent expansion of the Open Payments program to include advanced-practice clinicians (APCs) as covered recipients, we characterized the geographical distribution of general industry payments to nurse practitioners and physician assistants using the Open Payments database. The number and dollar value of payments, as well as the average and median payment amount earned per provider, varied by state. However, a significantly higher proportion of APCs received payments in states with more restrictive scope-of-practice laws. Understanding how and why payments to APCs vary by state can elucidate how industry–APC relationships are related to changing scope-of-practice and state-specific transparency/disclosure laws, informing future legislation.

## Introduction

Established as part of the Patient Protection and Affordable Care Act, the Open Payments program requires manufacturers to report payments and transfers of value made to covered recipients. While Open Payments initially only required payments made to physicians and teaching hospitals to be reported, the passage of the SUPPORT Act in 2018 expanded reporting requirements to include advanced-practice clinicians (APCs) such as nurse practitioners (NPs) and physician assistants (PAs).^1^ This change went into effect in 2021.

Due to state-specific scope-of-practice and transparency/disclosure laws, APCs in states granting greater autonomy may see more payments because they can make clinical decisions, such as prescriptions, independently with less physician oversight and input. Interestingly, we found that states with more restrictive scope-of-practice laws yielded significantly higher proportions of NPs (*P* < .0001) and PAs (*P* < .0001) receiving payments (Table 1). In this study, we use the Open Payments database to analyze nonresearch industry payments to characterize the geographical distribution of payments to NPs/PAs.

**Table 1.** Comparison of proportions of advanced-practice clinicians receiving payments by scope-of-practice law.

Scope-of-practice level (1 = least restrictive)	Mean (median) payment amount earned per-provider, $	Providers receiving payments, No.	Proportion of providers receiving payments, %	*P* value
Nurse practitioners				
1	498.47 (100.32)	37 199	39.57	<.0001
2	564.78 (120.17)	48 724	46.01	
3	534.97 (119.36)	71 476	56.11	
Physician assistants				
1	501.02 (117.55)	5858	25.59	<.0001
2	564.78 (124.80)	41 071	26.58	
3	534.97 (124.00)	24 431	30.03	

Source: Authors’ analysis of data from the Center of Medicare Services Open Payments program (General Payment Data, 2021). Nurse practitioners (NPs) are classified based on whether the state in which an NP practices in has granted full practice (level 1), partial practice (level 2), or restricted practice (level 3). Physician assistants (Pas) are classified based on whether the state in which a PA practices in has granted collaborative arrangement (level 1), physician supervision without co-signature (level 2), or physician supervision with co-signature required (level 3). States with more restrictive laws saw a greater proportion of NPs (*P* < .0001) and PAs (*P* <.0001) receiving payments.

## Data and methods

We performed a retrospective, cross-sectional study of non–research-related general industry payments to APCs in the United States using the 2021 Open Payments database. General payments include payments and transfers of value made to covered recipients by group purchasing organizations (GPOs), such as pharmaceutical and medical device companies. All data are submitted by GPOs and verified by Open Payments. This study was exempt from the Yale University Institutional Review Board because all data are publicly available.

Our analysis focused on payments made to NPs/PAs as they comprise the majority of total payments made to all APC groups.^2^ We aggregated the number and dollar value of payments to each APC's National Provider Identifier (NPI) number to identify the number of providers receiving payments. We further aggregated these payments by the provider's license-issuing state and included the District of Columbia in our analysis. APCs practicing in US territories were excluded. Payment data were linked by NPI to the National Plan and Provider Enumeration System database for gender classification and estimates of total providers per state.

State-level descriptive statistics were tabulated for NPs/PAs separately and include the total number/dollar value of payments, mean/median payment amounts earned per provider, which were calculated out of all NPs/PAs receiving payments in that state, percentage of APCs receiving payments, percentage of female APCs receiving payments, proportion of total payments earned by females, and the distribution of payments by payment type.

Differences in the proportion of APCs receiving payments according to scope-of-practice level were analyzed using the chi-square and Fisher's exact test. For NPs, states were classified as full practice (level 1), where physician supervision is not required; partial practice (level 2), where NPs are partially restricted from independently practicing their scope-of-practice; or restricted practice (level 3), which requires physician supervision for all scope-of-practice.^2,3^ For PAs, states were classified as collaborative arrangement (level 1), characterized as a less hierarchical physician–PA relationship without formal oversight, physician supervision without co-signature (level 2), or physician supervision with co-signature required (level 3).^2,4^

## Results

In 2021, 157 399 NPs received 2 338 765 payments valued at $78 844 108, and 70 433 PAs received 987 348 payments valued at $31 134 227. In most states, female APCs earned a greater proportion of payments than males (Tables 2 and 3). While there is no clear relationship between mean/median payment amounts per provider and scope-of-practice laws, states allowing APCs greater autonomy saw a significantly lower proportion of APCs receiving payments (Table 1). Rural states,^5^ such as Maine or Alaska, tend to allow APCs greater autonomy but also have lower proportions of APCs with payments and payment totals (Tables 2 and 3).

**Table 2.** Characteristics of industry payments to nurse practitioners by state in 2021.

State	Scope-of-practice level (1 = least restrictive)	No. of payments	Total payment amount, $	No. of NPs receiving payments (% of all state providers)	Percent of female NPs (% of total payments to females)	Mean (median) payment amount earned per NP, $
Alabama	2	91 888	2 040 008.55	4250 (74.84)	86.26 (88.66)	480.00 (130.34)
Alaska	1	3125	171 415.59	366 (45.86)	83.61 (76.22)	468.35 (90.87)
Arizona	1	64 903	2 496 863.68	4093 (51.00)	82.90 (82.35)	610.03 (131.18)
Arkansas	2	37 787	1 093 043.97	2322 (56.79)	86.11 (55.97)	470.73 (113.35)
California	3	125 433	5 732 854.09	9522 (71.71)	83.93 (80.95)	602.06 (114.26)
Colorado	1	23 475	1 031 991.95	2327 (47.87)	90.14 (80.17)	443.49 (92.38)
Connecticut	1	28 837	1 374 930.04	2044 (53.80)	88.85 (90.82)	672.67 (109.05)
Delaware	1	10 317	285 048.25	781 (62.83)	87.91 (92.85)	364.98 (110.32)
District of Columbia	1	4837	324 336.48	636 (78.62)	90.63 (93.57)	509.96 (83.14)
Florida	3	223 260	7 671 228.93	14 938 (64.97)	86.26 (89.33)	513.54 (124.52)
Georgia	3	107 382	3 064 465.10	6790 (69.69)	88.10 (84.81)	451.32 (114.82)
Hawaii	1	5763	191 218.55	400 (56.02)	85.11 (81.66)	478.05 (125.00)
Idaho	1	9897	439 184.73	857 (42.47)	75.81 (90.44)	512.47 (90.86)
Illinois	2	66 710	2 385 078.21	5155 (38.10)	91.35 (92.46)	462.67 (107.24)
Indiana	2	81 460	2 423 255.56	4373 (42.10)	92.04 (87.92)	554.14 (129.31)
Iowa	1	20 849	865 067.37	1696 (34.49)	92.65 (90.44)	510.06 (97.70)
Kansas	2	30 749	963 922.82	2181 (50.83)	91.40 (92.79)	441.96 (110.80)
Kentucky	1	80 453	2 476 180.36	4654 (51.72)	90.03 (93.77)	532.05 (115.52)
Louisiana	2	67 363	2 146 754.73	3647 (65.42)	83.27 (87.31)	588.64 (129.69)
Maine	1	3120	93 490.50	425 (18.64)	93.98 (91.98)	219.98 (93.38)
Maryland	1	33 798	1 316 551.92	2900 (40.84)	89.90 (93.11)	453.98 (99.20)
Massachusetts	1	24 589	1 144 556.39	2512 (29.89)	91.34 (83.30)	455.64 (98.18)
Michigan	3	51 877	1 768 233.11	3933 (41.18)	87.86 (85.35)	449.59 (118.57)
Minnesota	1	3529	299 254.78	867 (13.46)	90.79 (75.22)	345.16 (41.58)
Mississippi	2	65 243	1 604 978.59	3323 (62.50)	86.55 (86.40)	482.99 (121.20)
Missouri	3	56 963	1 473 588.07	3451 (40.40)	92.08 (94.07)	427.00 (118.92)
Montana	1	2244	103 407.01	360 (29.73)	84.66 (92.74)	287.24 (53.89)
Nebraska	1	15 825	574 001.95	1120 (45.71)	92.15 (93.93)	512.50 (103.17)
Nevada	1	19 740	747 428.85	1328 (55.68)	81.00 (61.83)	562.82 (123.75)
New Hampshire	1	4238	216 112.25	446 (21.28)	91.85 (70.34)	484.56 (100.66)
New Jersey	1	47 682	1 615 722.16	4046 (56.09)	88.74 (85.86)	399.34 (92.48)
New Mexico	1	8277	266 634.74	918 (43.02)	80.34 (81.03)	290.45 (92.75)
New York	2	110 767	3 991 763.11	8576 (56.19)	88.85 (84.97)	465.46 (124.94)
North Carolina	3	83 555	2 508 556.82	5070 (45.59)	88.86 (83.89)	494.78 (115.00)
North Dakota	1	2986	150 720.00	387 (28.99)	89.63 (94.08)	389.46 (74.81)
Ohio	2	107 860	3 503 882.42	7297 (42.99)	88.53 (84.34)	480.18 (114.26)
Oklahoma	3	26 033	563 682.48	1703 (48.28)	88.45 (89.78)	330.99 (107.67)
Oregon	1	6970	338 295.78	834 (22.83)	84.97 (89.80)	405.63 (59.09)
Pennsylvania	2	72 902	3 428 919.19	4900 (38.94)	89.10 (92.93)	699.78 (115.69)
Rhode Island	1	5583	233 513.22	365 (33.18)	89.14 (84.93)	639.76 (112.96)
South Carolina	3	50 710	1 254 312.50	2887 (52.91)	90.04 (86.29)	434.47 (120.44)
South Dakota	2	3824	144 120.22	395 (23.62)	89.91 (40.33)	364.86 (88.69)
Tennessee	3	124 428	3 801 455.28	6417 (55.21)	87.69 (86.17)	592.40 (124.99)
Texas	3	202 173	5 681 944.13	13 035 (55.58)	84.38 (87.08)	435.90 (120.81)
Utah	1	19 504	997 461.33	1177 (52.76)	68.63 (59.17)	847.46 (125.00)
Vermont	1	36	3359.33	23 (2.46)	89.47 (63.59)	146.06 (73.44)
Virginia	3	53 234	1 900 530.57	3730 (46.01)	91.87 (78.68)	509.53 (107.62)
Washington	1	12 796	750 096.97	1438 (22.83)	83.94 (82.51)	521.63 (77.38)
West Virginia	2	20 606	576 072.85	1137 (41.47)	90.74 (85.35)	506.66 (106.80)
Wisconsin	2	11 388	578 746.43	1168 (15.00)	92.05 (92.33)	495.50 (100.00)
Wyoming	1	1797	35 866.39	199 (35.86)	94.34 (95.43)	180.23 (77.76)

Source: Authors’ analysis of data from the Center of Medicare Services Open Payments program (General Payment Data, 2021). The mean and median values are calculated out of all NPs receiving payments in that state. States were classified as full practice (1), where physician supervision is not required; partial practice (2), where NPs are restricted from practicing independently for some aspects of their scope of practice; or restricted practice (3), which requires physician supervision for all scope of practice. Abbreviation: NP, nurse practitioner.

**Table 3.** Characteristics of industry payments to physician assistants by state in 2021.

State	Scope-of-practice level (1 = least restrictive)	No. of payments	Total payment amount, $	No. of PAs receiving payments (% of all state providers)	Percent of female PAs (% of total payments to females)	Mean (median) payment amount earned per PA, $
Alabama	3	12 964	369 638.05	730 (54.03)	64.40 (83.45)	506.35 (126.52)
Alaska	1	2905	79 926.94	266 (33.59)	50.90 (38.04)	300.48 (115.86)
Arizona	2	39 588	1 318 677.29	2142 (36.12)	66.67 (51.26)	615.63 (138.81)
Arkansas	2	8211	328 835.76	398 (56.61)	60.16 (44.18)	826.22 (138.28)
California	3	102 003	3 682 680.11	6039 (46.75)	64.22 (64.38)	609.82 (124.96)
Colorado	3	26 146	1 118 173.50	1858 (29.18)	69.62 (70.80)	601.82 (110.44)
Connecticut	2	15 896	484 556.92	1157 (24.72)	69.97 (67.26)	418.80 (110.43)
Delaware	2	4578	122 583.58	395 (30.29)	57.48 (46.47)	310.34 (98.94)
District of Columbia	2	2331	75 221.94	204 (53.83)	89.09 (96.04)	368.74 (99.05)
Florida	2	103 448	4 384 626.28	5947 (36.95)	68.53 (69.56)	737.28 (161.96)
Georgia	2	53 277	2 053 685.35	3028 (47.34)	65.81 (59.27)	678.23 (110.80)
Hawaii	3	2150	60 103.36	134 (35.73)	60.99 (71.94)	448.53 (125.52)
Idaho	2	8922	241 233.50	684 (24.52)	44.85 (29.30)	352.68 (105.22)
Illinois	1	35 682	1 112 053.82	2177 (26.42)	75.61 (71.04)	510.82 (122.12)
Indiana	2	14 769	411 712.35	919 (21.51)	70.39 (68.45)	448.00 (122.28)
Iowa	3	8806	366 246.81	664 (25.66)	66.70 (67.51)	551.58 (112.99)
Kansas	3	15 260	379 127.91	740 (36.01)	70.96 (68.66)	512.34 (136.23)
Kentucky	3	18 785	554 199.02	906 (33.57)	70.16 (66.32)	611.70 (155.91)
Louisiana	3	15 621	513 879.93	946 (44.41)	70.33 (70.45)	543.21 (133.98)
Maine	2	1228	51 219.93	198 (10.23)	62.94 (60.59)	258.69 (82.73)
Maryland	2	23 889	776 321.61	1586 (26.40)	68.73 (72.79)	489.48 (106.62)
Massachusetts	2	11 879	497 836.28	1093 (17.42)	70.67 (62.44)	455.48 (99.00)
Michigan	1	44 515	1 464 239.85	2555 (22.87)	67.79 (48.09)	573.09 (120.04)
Minnesota	2	2869	215 499.58	683 (7.90)	69.57 (64.02)	315.52 (53.64)
Mississippi	2	5992	138 555.79	243 (51.27)	61.39 (78.21)	570.19 (224.30)
Missouri	3	9132	239 421.05	593 (18.51)	67.15 (58.59)	403.75 (95.00)
Montana	3	2007	109 140.09	270 (16.39)	57.28 (68.70)	404.22 (90.38)
Nebraska	3	11 381	251 073.61	723 (30.96)	69.64 (67.77)	347.27 (105.45)
Nevada	3	12 907	379 892.51	684 (42.90)	59.07 (52.75)	555.40 (173.38)
New Hampshire	2	1149	44 086.28	185 (10.57)	67.25 (73.73)	238.30 (103.25)
New Jersey	3	27 608	817 291.70	2013 (45.73)	73.04 (62.20)	406.01 (107.64)
New Mexico	1	3059	70 700.00	346 (28.38)	57.12 (58.44)	204.34 (81.79)
New York	2	100 038	3 300 305.49	6761 (31.59)	65.29 (69.25)	488.14 (124.99)
North Carolina	2	85 983	2 418 398.30	4078 (26.74)	67.29 (68.81)	593.04 (136.07)
North Dakota	2	1318	44 511.57	152 (19.56)	73.43 (37.30)	292.84 (64.46)
Ohio	3	25 777	776 558.31	1773 (20.00)	67.44 (66.40)	437.99 (108.41)
Oklahoma	2	17 796	468 479.24	957 (36.36)	66.31 (67.81)	489.53 (117.79)
Oregon	2	5884	179 937.59	665 (15.37)	62.50 (55.63)	270.58 (82.20)
Pennsylvania	3	71 792	1 949 150.18	3859 (19.15)	77.61 (61.89)	505.09 (125.03)
Rhode Island	2	2868	72 279.75	157 (21.07)	70.65 (75.37)	460.38 (110.18)
South Carolina	3	23 599	643 030.00	1099 (28.16)	70.10 (64.22)	585.10 (168.45)
South Dakota	2	1912	37 691.30	169 (14.47)	71.12 (56.00)	223.03 (50.70)
Tennessee	3	30 856	859 846.41	1392 (33.43)	66.12 (64.61)	617.71 (131.20)
Texas	2	111 045	3 350 738.78	5163 (35.61)	70.19 (60.08)	648.99 (164.23)
Utah	2	13 846	522 546.29	782 (23.68)	36.27 (49.77)	668.22 (127.01)
Vermont	3	13	313.16	8 (1.31)	73.53 (79.11)	39.15 (29.63)
Virginia	2	26 209	795 457.18	1680 (26.31)	71.82 (60.07)	473.49 (121.10)
Washington	2	9064	493 028.79	887 (11.45)	56.87 (44.42)	555.84 (71.75)
West Virginia	1	9576	208 056.17	514 (35.06)	65.59 (70.64)	404.78 (113.62)
Wisconsin	2	5288	348 544.05	641 (7.84)	75.19 (72.14)	543.75 (102.10)
Wyoming	2	978	20 219.77	117 (25.88)	52.29 (54.73)	172.82 (60.16)

Source: Authors’ analysis of data from the Center of Medicare Services Open Payments program (General Payment Data, 2021). The mean and median values are calculated out of all PAs receiving payments in that state. States were classified as collaborative arrangement (1), characterized as a less hierarchical physician–PA relationship without formal oversight; physician supervision without co-signature (2); or physician supervision with co-signature required (3). Abbreviation: PA, physician assistant.

Payment data for APCs are positively skewed in all states. Although food and beverage payments account for over 90% of the number of payments to APCs (Appendix Exhibits S1 and S2),^6^ they typically account for 45%–75% of each state's total payment value (Tables 2 and 3). Payments for non–consulting services may comprise only 1%–2% of the number of payments, but they can account for 15%–45% of each state's total payment value. States with higher mean per-provider payment values tend to see a greater proportion of payments derived from payments for non–consulting services.

**Figure 1.**
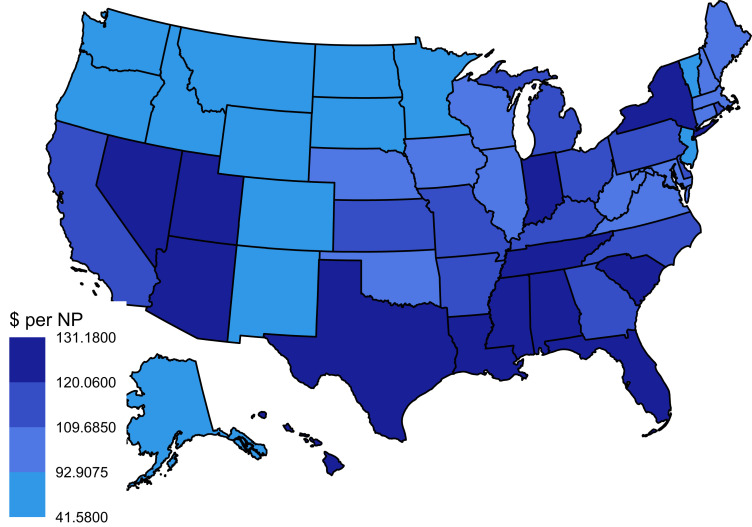
Differences in median payment amount earned per nurse practitioner (NP) by state. Source: Authors’ analysis of data from the Center of Medicare Services Open Payments program (General Payment Data, 2021). Each state is color-coded by the median payment value earned per NP for that state. Each color corresponds to a quartile; darker colors correspond to a higher quartile and median value.

**Figure 2.**
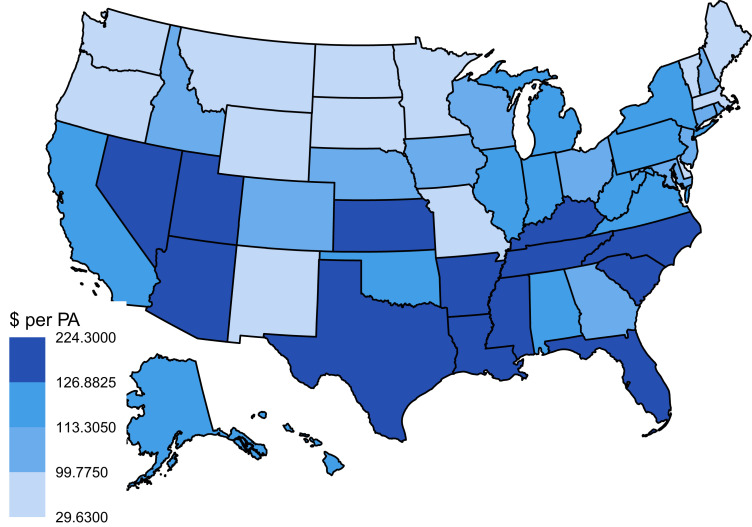
Differences in median payment amount earned per physician assistant (PA) by state. Source: Authors’ analysis of data from the Center of Medicare Services Open Payments program (General Payment Data, 2021). Each state is color-coded by the median payment value earned per PA for that state. Each color corresponds to a quartile; darker colors correspond to a higher quartile and median value.

In general, states with the highest median value of payments were concentrated in the southern/western regions. For NPs, 7 states in the South (Alabama, Florida, Louisiana, Mississippi, South Carolina, Tennessee, and Texas) and 4 states in the West (Arizona, Hawaii, Nevada, and Utah) saw medians in the top quartile of all states, whereas the Midwest/Northeast regions each had 1 state with a median in the top quartile (Indiana and New York, respectively) (Figure 1). For PAs, 9 states in the South (Arkansas, Florida, Kentucky, Louisiana, Mississippi, North Carolina, South Carolina, Tennessee, and Texas) and 3 states in the West (Arizona, Nevada, and Utah) had medians in the top quartile, whereas 1 state from the Midwest/Northeast had a state with a median in the top quartile (Kansas) (Figure 2).

## Discussion

General industry payments to APCs in 2021 were predominantly earned by females in most states and differentially distributed between states, in total number and value of payments but also in the mean and median values of payments earned per provider. States with more restrictive practice laws saw a greater proportion of APCs receiving payments, and states with the highest median payment values were concentrated in the southern/western United States. The median payment amounts earned per NP/PA are within the IQR of the median amounts earned per physician ($167; IQR = $45–$710).^2^ This study presents comprehensive data on industry payments to APCs, focusing specifically on differences in payments at the state level.

Our study contains limitations. First, we cannot discount inherent reporting and identification errors made by Open Payments or delayed submissions by GPOs. Payments in the form of free samples are also neither reported nor quantified. Second, APCs receiving payments who practice in multiple states may be counted in each of those states, and their total payments may be divided between those states. Nevertheless, Open Payments remains the most comprehensive and reliable database for understanding industry–healthcare provider relationships.

The exemption of APCs from disclosure requirements has long been recognized as a limitation of Open Payments.^7^ Consequently, the nature and extent of industry–APC relationships are largely unknown. Despite their growing role in the healthcare system, APCs often fall “under the radar”^8^ in the policy climate as most transparency legislation has largely focused on physicians, which may make APCs susceptible targets by industry marketing efforts. Thus, the inclusion of APCs in Open Payments removes a loophole that the industry could previously exploit unnoticed.

Interpreting industry–APC relationships should incorporate an understanding of the geographic distribution of payments because the practice authority and legal context of receiving payments are state specific.^9–11^ States granting APCs greater autonomy have been hypothesized to receive more payments,^2^ but we found the opposite effect (Table 1). One possible explanation is the time course in policy change. States that have granted APCs more independence earlier may be associated with higher payments. For example, Arizona was a full-practice state for NPs before 2014,^12^ is one of the most progressive states in granting NPs greater independence,^4^ and has the highest median payment amount per NP (Table 2). States that have only recently expanded APCs’ scope-of-practice may not see any changes in payment data yet. Additionally, the observation that rural states tend to grant APCs more autonomy but yield the lowest per-provider payment values suggests that there is no absolute pattern, and the variations in payments by state cannot be attributable to scope-of-practice laws alone.

Vermont, Minnesota, Massachusetts, and the District of Columbia consistently yielded low median payment values for NPs/PAs (Tables 2 and 3).^6^ These states have explicitly included APCs as covered recipients in their state-specific transparency laws and imposed varying levels of bans on gifts and food items, with Vermont imposing a complete ban.^13^ Although we cannot conclude that these variations are caused by transparency laws, they are consistent with physician payment data that states with payment-restriction policies are associated with lower median payments.^14^ They also suggest that merely disclosing a financial relationship with the industry does not deter the formation of them. Public awareness of Open Payments and whether their provider received industry payments is low.^15^ However, the transparency laws in Vermont and Minnesota could serve as models for policymakers looking to balance the state practice environment with appropriate mechanisms to regulate undue industry influence on APCs.

In conclusion, we find that industry payments to APCs vary by state and may be modulated by factors including, but not limited to, scope-of-practice laws, rurality, types of payments received, geographic region, and transparency laws. Clinicians and policymakers should understand the unique legislative, legal, and geographical context of their state and clinical practice to understand how industry relationships might affect APCs, which can inform future legislation.

## Supplementary Material

qxad011_Supplementary_Data
