# Supplementary material for: Geographic distribution of general industry payments to advanced-practice clinicians
Source: Health Aff Sch. 2023 Jun 20;1(1):qxad011. doi: 10.1093/haschl/qxad011 (PMC10986193; doi:10.1093/haschl/qxad011)
Supplement: qxad011_Supplementary_Data [file qxad011_Supplementary_Data.zip › Appendix A1, A2.docx]

Appendix Exhibit A1. Distribution of Industry Payments to NPs by Type

| **State** | **% of Total Payment Value for Food/Beverage** | **% of Total Payment Value for Non-consulting Services** | **% of Total Payment Value for Consulting** | **% of Total Number of Payments for Food/Beverage** | **% of Total Number of Payments for Non-consulting Services** | **% of Total Payment Value for Consulting** |
| --- | --- | --- | --- | --- | --- | --- |
| AK | 41.50 | 44.82 | 5.38 | 95.36 | 0.19 | 1.82 |
| AL | 76.63 | 16.01 | 2.70 | 98.70 | 0.08 | 0.50 |
| AR | 59.93 | 30.77 | 3.57 | 97.98 | 0.09 | 0.64 |
| AZ | 54.63 | 32.30 | 6.55 | 96.60 | 0.29 | 1.12 |
| CA | 48.31 | 36.53 | 7.02 | 95.56 | 0.30 | 1.56 |
| CO | 47.28 | 37.18 | 8.95 | 95.92 | 0.40 | 1.89 |
| CT | 43.98 | 25.99 | 23.14 | 96.61 | 0.30 | 1.19 |
| DC | 35.89 | 50.50 | 8.84 | 93.18 | 0.68 | 3.29 |
| DE | 71.68 | 15.57 | 4.32 | 98.20 | 0.08 | 0.28 |
| FL | 62.71 | 27.07 | 4.24 | 97.59 | 0.14 | 0.75 |
| GA | 65.32 | 16.72 | 12.85 | 98.23 | 0.21 | 0.48 |
| HI | 72.94 | 12.85 | 1.28 | 97.26 | 0.07 | 0.57 |
| IA | 40.84 | 43.68 | 5.47 | 95.25 | 0.26 | 2.00 |
| ID | 42.85 | 44.58 | 3.51 | 95.57 | 0.18 | 1.41 |
| IL | 55.08 | 30.02 | 7.16 | 95.81 | 0.37 | 1.49 |
| IN | 60.02 | 28.84 | 3.52 | 97.39 | 0.13 | 0.83 |
| KS | 58.73 | 28.71 | 2.75 | 97.36 | 0.15 | 0.89 |
| KY | 55.53 | 34.64 | 2.73 | 97.61 | 0.12 | 0.82 |
| LA | 57.86 | 20.31 | 17.04 | 98.33 | 0.10 | 0.63 |
| MA | 47.06 | 37.07 | 10.49 | 95.45 | 0.43 | 1.79 |
| MD | 52.00 | 34.29 | 6.80 | 96.83 | 0.29 | 1.08 |
| ME | 75.87 | 11.99 | 3.86 | 96.83 | 0.06 | 0.29 |
| MI | 58.16 | 26.86 | 6.73 | 96.97 | 0.20 | 0.98 |
| MN | 25.87 | 44.14 | 16.17 | 87.45 | 1.73 | 4.70 |
| MO | 70.66 | 20.76 | 2.18 | 97.88 | 0.07 | 0.57 |
| MS | 70.05 | 22.27 | 1.34 | 98.37 | 0.05 | 0.63 |
| MT | 41.21 | 45.80 | 5.47 | 94.83 | 0.67 | 1.87 |
| NC | 59.46 | 28.98 | 5.17 | 97.60 | 0.17 | 0.80 |
| ND | 34.63 | 26.38 | 11.65 | 95.78 | 0.84 | 0.90 |
| NE | 45.41 | 43.89 | 1.06 | 96.77 | 0.12 | 1.11 |
| NH | 41.18 | 35.85 | 13.18 | 94.64 | 0.90 | 1.49 |
| NJ | 55.61 | 28.58 | 7.80 | 96.82 | 0.36 | 0.91 |
| NM | 65.51 | 21.45 | 6.92 | 97.27 | 0.24 | 1.03 |
| NV | 55.88 | 25.07 | 9.59 | 96.00 | 0.50 | 0.81 |
| NY | 63.38 | 25.21 | 4.85 | 97.13 | 0.29 | 0.73 |
| OH | 57.40 | 31.77 | 4.80 | 97.17 | 0.17 | 0.92 |
| OK | 82.75 | 7.07 | 2.95 | 98.82 | 0.07 | 0.22 |
| OR | 38.30 | 38.52 | 13.05 | 93.82 | 0.93 | 1.62 |
| PA | 41.81 | 40.18 | 9.05 | 95.55 | 0.38 | 1.52 |
| RI | 51.35 | 40.52 | 5.04 | 96.51 | 0.47 | 1.59 |
| SC | 73.46 | 18.65 | 3.19 | 98.49 | 0.06 | 0.45 |
| SD | 49.08 | 28.48 | 2.77 | 95.11 | 0.29 | 1.02 |
| TN | 59.20 | 31.08 | 3.06 | 97.69 | 0.10 | 0.96 |
| TX | 71.38 | 16.71 | 4.91 | 98.28 | 0.17 | 0.46 |
| UT | 32.30 | 47.44 | 7.59 | 94.26 | 0.45 | 2.43 |
| VA | 52.73 | 37.80 | 4.00 | 97.14 | 0.19 | 1.07 |
| VT | 29.75 | 31.11 | 7.44 | 75.00 | 2.78 | 2.78 |
| WA | 34.75 | 42.90 | 13.41 | 93.49 | 0.74 | 1.93 |
| WI | 43.16 | 36.12 | 9.75 | 93.84 | 0.98 | 1.89 |
| WV | 60.22 | 24.55 | 9.91 | 98.24 | 0.08 | 0.61 |
| WY | 86.85 | 0.05 | 6.51 | 97.55 | 0.39 | 0.06 |

SOURCE: Authors’ analysis of data from the Center of Medicare Services Open Payments program (General Payment Data, 2021).

NOTES: Calculated by state, the percentage of total number and value of payments accounted for by food and beverage, non-consulting services, and consulting payments, which are the top three payment types for which payments come from.

Appendix Exhibit A2. Distribution of Industry Payments to PAs by Type

| **State** | **% of Total Payment Value for Food/Beverage** | **% of Total Payment Value for Non-consulting Services** | **% of Total Payment Value for Consulting** | **% of Total Number of Payments for Food/Beverage** | **% of Total Number of Payments for Non-consulting Services** | **% of Total Payment Value for Consulting** |
| --- | --- | --- | --- | --- | --- | --- |
| AK | 79.43 | 1.81 | 7.80 | 97.80 | 0.17 | 0.24 |
| AL | 66.21 | 4.53 | 20.29 | 97.63 | 0.15 | 0.86 |
| AR | 43.02 | 6.08 | 38.47 | 96.08 | 0.17 | 1.18 |
| AZ | 58.30 | 5.30 | 26.31 | 97.23 | 0.22 | 0.82 |
| CA | 58.01 | 6.02 | 26.34 | 96.73 | 0.20 | 0.86 |
| CO | 49.36 | 6.83 | 31.29 | 96.21 | 0.30 | 1.35 |
| CT | 68.07 | 7.13 | 14.88 | 97.55 | 0.23 | 0.58 |
| DC | 65.32 | 8.00 | 14.14 | 96.14 | 0.43 | 1.20 |
| DE | 77.15 | 0.78 | NA | 97.97 | 0.04 | NA |
| FL | 51.40 | 9.82 | 27.62 | 96.75 | 0.24 | 0.97 |
| GA | 49.43 | 5.61 | 31.61 | 96.66 | 0.21 | 1.05 |
| HI | 83.04 | 1.23 | 7.40 | 98.09 | 0.05 | 0.33 |
| IA | 44.01 | 5.53 | 31.20 | 94.73 | 0.35 | 1.28 |
| ID | 66.29 | 2.72 | 13.66 | 97.83 | 0.12 | 0.39 |
| IL | 59.35 | 7.86 | 19.11 | 97.52 | 0.25 | 0.49 |
| IN | 67.04 | 4.84 | 18.30 | 97.72 | 0.16 | 0.60 |
| KS | 69.79 | 2.80 | 12.13 | 98.03 | 0.10 | 0.37 |
| KY | 57.30 | 9.98 | 25.17 | 97.83 | 0.36 | 0.54 |
| LA | 57.78 | 7.40 | 28.01 | 97.67 | 0.12 | 0.67 |
| MA | 53.37 | 7.56 | 23.56 | 95.67 | 0.45 | 1.14 |
| MD | 60.48 | 7.50 | 22.69 | 97.11 | 0.30 | 0.73 |
| ME | 56.54 | NA | 29.06 | 96.50 | NA | 0.16 |
| MI | 55.91 | 5.43 | 27.93 | 97.18 | 0.13 | 0.90 |
| MN | 32.45 | 18.59 | 9.41 | 89.16 | 2.09 | 0.73 |
| MO | 72.93 | 1.46 | 12.58 | 97.45 | 0.07 | 0.41 |
| MS | 78.50 | 0.14 | 7.70 | 98.35 | 0.03 | 0.27 |
| MT | 35.72 | 2.41 | 39.12 | 93.52 | 0.25 | 1.84 |
| NC | 62.34 | 4.41 | 25.87 | 97.85 | 0.15 | 0.67 |
| ND | 50.15 | 12.80 | 27.85 | 96.36 | 0.46 | 1.29 |
| NE | 71.83 | 3.59 | 15.31 | 98.45 | 0.07 | 0.26 |
| NH | 67.90 | 15.70 | 1.36 | 94.95 | 0.78 | 0.09 |
| NJ | 62.74 | 19.69 | 9.87 | 98.13 | 0.25 | 0.29 |
| NM | 92.93 | NA | 2.26 | 98.92 | NA | 0.07 |
| NV | 71.34 | 11.15 | 6.89 | 97.14 | 0.39 | 0.22 |
| NY | 67.26 | 3.92 | 20.19 | 97.62 | 0.17 | 0.59 |
| OH | 61.02 | 11.20 | 13.11 | 96.75 | 0.38 | 0.50 |
| OK | 69.69 | 4.83 | 15.05 | 98.29 | 0.13 | 0.36 |
| OR | 62.19 | 2.86 | 7.51 | 96.23 | 0.25 | 0.15 |
| PA | 69.14 | 5.52 | 14.10 | 97.78 | 0.20 | 0.41 |
| RI | 81.33 | 4.77 | 0.83 | 98.47 | 0.07 | 0.03 |
| SC | 70.27 | 7.91 | 13.14 | 98.16 | 0.18 | 0.44 |
| SD | 85.20 | NA | NA | 98.22 | NA | NA |
| TN | 63.97 | 3.21 | 26.16 | 97.73 | 0.10 | 0.58 |
| TX | 65.19 | 6.64 | 19.11 | 97.98 | 0.23 | 0.53 |
| UT | 44.05 | 6.51 | 38.81 | 96.35 | 0.34 | 1.00 |
| VA | 64.06 | 14.45 | 16.39 | 97.89 | 0.45 | 0.52 |
| VT | 69.68 | NA | NA | 92.31 | NA | NA |
| WA | 37.35 | 30.99 | 11.35 | 94.92 | 0.66 | 0.57 |
| WI | 36.29 | 27.07 | 22.11 | 93.17 | 1.25 | 1.49 |
| WV | 72.54 | 1.64 | 16.99 | 98.75 | 0.06 | 0.30 |
| WY | 73.91 | NA | NA | 95.81 | NA | NA |

SOURCE: Authors’ analysis of data from the Center of Medicare Services Open Payments program (General Payment Data, 2021).

NOTES: Calculated by state, the percentage of total number and value of payments accounted for by food and beverage, non-consulting services, and consulting payments, which are the top three payment types for which payments come from.
